# Welcoming new Editors to Disease Models & Mechanisms

**DOI:** 10.1242/dmm.053105

**Published:** 2026-07-22

**Authors:** Kirsty Hooper

**Affiliations:** The Company of Biologists, Bidder Building, Station Road, Histon, Cambridge CB24 9LF, UK

## Abstract

**Summary:** DMM broadens its editorial expertise by welcoming two new Editors to our team: Satdarshan (Paul) Singh Monga and Shinya Yamamoto.

**Figure:**
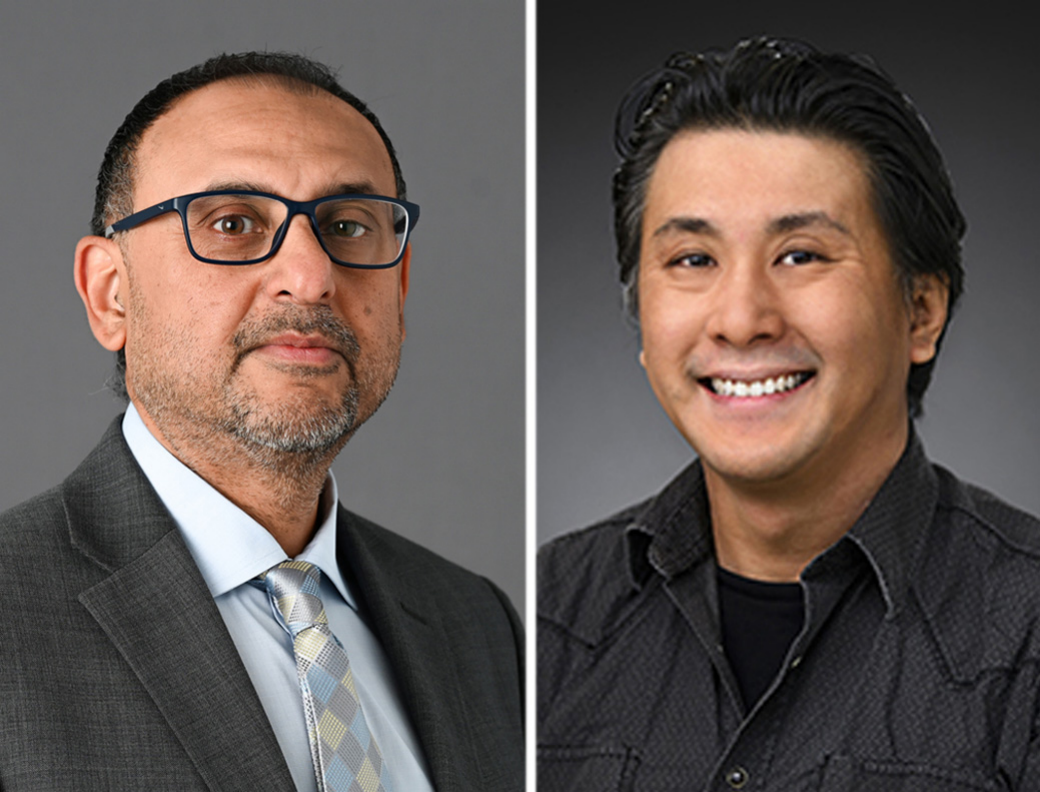
Satdarshan (Paul) Singh Monga (left) and Shinya Yamamoto (right)

At the heart of Disease Models & Mechanisms (DMM) is a dedicated team of research-active Editors who work to maintain our high standards in publishing cutting-edge disease-focused research, steer the direction of the journal within dynamic research ecosystems and strengthen our connections with numerous research communities. They are responsible for guiding each of our Research and Resources & Methods articles through the publication process and ensure that only high-quality and robust disease biology research is published in DMM. Owing to our multidisciplinary scope, we are proud to have appointed a diverse team of scientists with a broad range of expertise that spans fundamental biology and clinical practice (Box 1). To enrich this team further, we are happy to welcome two new members: Satdarshan (Paul) Singh Monga (University of Pittsburgh, Pittsburgh, PA, USA) and Shinya Yamamoto [Baylor College of Medicine (BCM), Houston, TX, USA]. Our team of expert academic editors is keen to handle your next submission. You can also explore examples of recently published DMM articles in Paul (Khan et al., 2024; Maggiore et al., 2025; May et al., 2025; VanSant-Webb et al., 2026) and Shinya's (Khan and Rusan, 2025; Link et al., 2024; Rogic et al., 2025; Verheyen, 2022) areas of expertise.

Box 1. Expertise of existing DMM Editors **Editor-in-Chief**

**E. Elizabeth Patton**

Liz Patton is a Professor and MRC Investigator at the MRC Human Genetics Unit, MRC Institute of Genetics and Molecular Medicine, University of Edinburgh, Edinburgh, UK. Liz's laboratory uses chemical genetic approaches in zebrafish to investigate the basis of melanoma biology and drug discovery, particularly focusing on melanocyte development and the neural crest.

**Deputy Editor-in-Chief**

**Elaine R. Mardis**

Elaine Mardis is co-Executive Director of the Institute for Genomic Medicine at Nationwide Children's Hospital and Professor of Pediatrics at the Ohio State University College of Medicine, Columbus, OH, USA. Her research interests focus on the application of next-generation sequencing to characterise cancer genomes and transcriptomes, and to support therapeutic decision making. Her translational research efforts include the development of sequencing-based diagnostics, decision-support tools and databases, and the use of genomics to design personalised cancer vaccines.

**Editors**

**James Amatruda**

James Amatruda is based at the Children's Hospital Los Angeles, Los Angeles, CA, USA, where he is Head of Basic and Translational Research in the Cancer and Blood Disease Institute and Professor of Pediatrics and Medicine at the University of Southern California Keck School of Medicine. His clinical specialty is the care of children with sarcomas, germ cell tumours, Wilms tumours and other paediatric solid tumours. His laboratory uses primary tumour genomics and zebrafish genetic models to better understand disease mechanisms and to promote new therapeutic approaches for childhood cancers.

**Steven J. Clapcote**

Steve Clapcote is a lecturer in Pharmacology in the School of Biomedical Sciences at the University of Leeds, Leeds, UK. His research applies mouse and human molecular genetics to investigate mechanisms of human neurological diseases, including psychiatric, neurodevelopmental, neurodegenerative and movement disorders, with the aim of identifying new targets for therapeutic intervention.

**Vivian Li**

Vivian Li is Assistant Research Director and a senior group leader at The Francis Crick Institute, London, UK. Using mouse models and organoids, her laboratory investigates the regulation of intestinal homeostasis and cancer, with primary focus on the WNT signalling pathway. Her laboratory has also pioneered the use of organoids to engineer laboratory-grown human intestinal grafts, with potential use as an innovative replacement therapy to treat intestinal failure in the future.

**Karen Liu**

Karen Liu is Professor of Genetics and Development in the Centre for Craniofacial and Regenerative Biology at King's College London, London, UK. Karen's laboratory uses mouse, frog and human stem cell models combined with chemical genetic approaches to study the role of cilia and neural crest development in congenital anomalies and cancers (neuroblastoma).

**Owen J. Sansom**

Owen Sansom is Director of the Cancer Research UK Beatson Institute in Glasgow, UK. His laboratory investigates two types of epithelial tumour – colorectal and pancreatic cancers. The focus of Owen's group is to model common mutational drivers within the mouse to identify novel markers of the disease, as well as targets for therapy. Over the past 5 years, his work has defined potential new therapeutic targets for colorectal cancers lacking APC and for resectable pancreatic cancer.

**David M. Tobin**

David Tobin works in the Departments of Molecular Genetics, and Microbiology and Immunology at Duke University School of Medicine, Durham, NC, USA. His research focuses largely on tuberculosis, innate immunity, mycobacterial pathogenesis and the host response to mycobacterial infection. Using a zebrafish model, his laboratory has identified new host susceptibility loci for mycobacterial infection and translated these findings into human cohorts.

**Associate Editors**

**Sally L. Dunwoodie**

Sally Dunwoodie leads the Embryology Laboratory and the Congenital Heart Disease Research Program at the Victor Chang Cardiac Research Institute, Darlinghurst, NSW, Australia. She is also a Professor in the Faculty of Medicine at the University for New South Wales, Sydney, NSW, Australia. Her research focuses on understanding developmental mechanisms that drive mammalian embryogenesis and on identifying disruptive genetic and environmental factors. With an emphasis on heart and vertebral formation, her research combines patient genome sequencing with mouse models of congenital malformation to study the impact of gestational stresses such as hypoxia and nutrition.

**Monkol Lek**

Monkol Lek is Associate Professor in the Department of Genetics at Yale School of Medicine, New Haven, CT, USA. His research is focused on the genetics of rare diseases, including identifying causal genes/variants, disease mechanisms and gene editing. His main area of interest is rare muscle disorders, and he has strong expertise in computational biology.

**Ian Smyth**

Ian Smyth is Associate Dean for Research and Research Infrastructure in the Faculty of Medicine at Monash University and Head of the Developmental Nephrology Group at the Monash Biomedicine Discovery Institute, Clayton, VIC, Australia. His group applies mouse genetics and disease modelling to address how development of the foetal renal collecting duct system is impacted by genetic mutation and environmental perturbation, and how disruption of signalling in these cells in the adult kidney gives rise to cysts in patients with ciliopathy.

**Eckhard Wolf**

Eckhard Wolf is a trained veterinarian and Director of the Center for Innovative Medical Models (CiMM), Ludwig Maximilian University of Munich, Munich, Germany. His laboratory specialises in the generation, characterisation and implementation of genetically engineered pigs as disease (diabetes mellitus, rare monogenic diseases) models and as organ donors for xenotransplantation.

## Satdarshan (Paul) Singh Monga

Paul Monga is the Senior Vice Chancellor Endowed Chair in Pathobiology and Therapeutics and Professor of Pharmacology and of Medicine at the University of Pittsburgh. He is also the founding director of the Pittsburgh Liver Research Centre and Inaugural Director of the Organ Pathobiology and Therapeutics Institute. Paul is a physician scientist, having trained in medicine at Dayanand Medical College and Hospital, Ludhiana, India, prior to completing postdoctoral fellowships at Georgetown University, Washington, DC, USA; Temple University, Philadelphia, PA, USA; and University of Pittsburgh. In 2001, he established his laboratory to study the cellular and molecular mechanisms of liver development, metabolism, regeneration, fibrosis and tumorigenesis. Paul's laboratory is a driving force behind the progression of personalised therapeutics for liver pathologies, with two clinical trials currently running that were built upon research in his laboratory using mouse models to develop therapies targeting the Wnt/β-catenin signalling pathway in liver cancer (Adebayo Michael et al., 2019; Lehrich et al., 2025; ClinicalTrials.gov IDs NCT06811116 and  NCT06600321). Among many awards, Paul was recognised with the Takeda Distinguished Research Award in the Gastrointestinal and Liver Physiology Section from the American Physiological Society in 2019 and the Outstanding Investigator Award by the American Society of Investigative Pathology in 2013 and 2014. In 2025, Paul served as President of the American Society of Investigative Pathology. He has also been elected into the American Society for Clinical Investigation and the Association of American Physicians. Paul's innovative use of mouse modelling and integration with the clinic to address pressing challenges in liver pathobiology will ensure that DMM remains at the forefront of translational research in this area.

## Shinya Yamamoto

Shinya Yamamoto is Associate Professor in the Department of Molecular and Human Genetics and Department of Neuroscience at BCM, and an Investigator at Jan and Dan Duncan Neurological Research Institute (NRI) at Texas Children's Hospital, Houston, TX, USA. Shinya originally trained as a veterinarian at the University of Tokyo Veterinary Medical Centre, Tokyo, Japan. He then did a PhD with renowned geneticist and *Drosophila* researcher Hugo Bellen at BCM (Bellen, 2022). Shinya proceeded to an independent research fellow position at the NRI and established his laboratory there. His laboratory continues to pioneer the use of *Drosophila* to study various diseases of the nervous system, including rare neurodevelopmental disorders (Yamamoto et al., 2014), autism (Marcogliese et al., 2022), Alzheimer's disease (Salazar et al., 2021) and infectious diseases, such as zika virus (Link et al., 2024) and SARS-CoV-2 (Guichard et al., 2023). Shinya is also one of the co-directors of the *Drosophila* Core for the Model Organisms Screening Center (MOSC) for the Undiagnosed Diseases Network (UDN) and serves on the Executive Committee of the UDN. Working with the UDN and other clinical research programmes, such as the GREGoR (Genomics Research to Elucidate the Genetics of Rare diseases) Consortium, his laboratory uses fruit flies to undertake functional genetics studies for rare and ultra-rare diseases to gain mechanistic insights into the diseases and help patients obtain more accurate genetic diagnoses (Yamamoto et al., 2024). His work on rare diseases expanded further through his involvement in the development of novel and vital computational tools, such as MARRVEL (Wang et al., 2017), AI-MARRVEL (Mao et al., 2024) and ModelMatcher (Harnish et al., 2022). In 2025, he was awarded the Genetics Society of America Early Career Medal for his innovative use of *Drosophila* to study human genetics, rare disease and neurology. Such expertise will also strengthen DMM's ongoing commitment to these research communities.
